# Evaluation of Anti-Xa Target Attainment with Prophylactic Enoxaparin Dosing Regimens for Venous Thromboembolism Prophylaxis in Morbidly Obese Patients

**DOI:** 10.3390/pharmacy12050133

**Published:** 2024-08-28

**Authors:** Andrew Sabers, Emilie Langenhan, Sean N. Avedissian, Brandon Reynolds

**Affiliations:** 1 Department of Pharmaceutical Care, University of Iowa Hospitals and Clinics, 200 Hawkins Dr, Iowa City, IA 52242, USA; 2 Department of Pharmaceutical and Nutrition Care, University of Nebraska Medical Center, 981090 Nebraska Medical Center, Omaha, NE 68198, USA; elangenhan@nebraskamed.com (E.L.); brreynolds@nebraskamed.com (B.R.); 3 College of Pharmacy, University of Nebraska Medical Center, 986145 Nebraska Medical Center, Omaha, NE 68198, USA; sean.avedissian@unmc.edu

**Keywords:** enoxaparin, obesity, venous thromboembolism

## Abstract

Subcutaneous enoxaparin has been shown to reduce the risk of venous thromboembolism (VTE) among hospitalized patients. However, alternative enoxaparin dosing strategies may be necessary in morbid obesity. The objective of this study was to assess the rate of target anti-Xa attainment with three enoxaparin dosing regimens for venous thromboembolism (VTE) prophylaxis in morbidly obese patients. In this retrospective study, anti-Xa target attainment was assessed among adult patients with a body mass index (BMI) ≥ 40 kg/m^2^ receiving enoxaparin 40 mg twice daily (BID), 60 mg BID, or 0.5 mg/kg BID. Univariate and multivariate analyses were conducted. Target anti-Xa levels were defined as a steady-state, peak level of 0.2–0.5 IU/mL. This study included 120 patients with 55 patients receiving 40 mg BID, 44 patients receiving 60 mg BID, and 21 patients receiving 0.5 mg/kg BID. Target anti-Xa levels were achieved in 29.1% of patients in the 40 mg BID arm, 54.5% in the 60 mg BID arm, and 90.5% in the 0.5 mg/kg BID arm. Anti-Xa target attainment was significantly increased in both the 60 mg BID arm (*p* = 0.01) and the 0.5 mg/kg arm (*p* < 0.0001), compared to the 40 mg BID arm. In morbidly obese patients, weight-based dosing was associated with a greater attainment of target anti-Xa levels. Further studies are needed to determine the impact of these dosing regimens on clinical outcomes.

## 1. Introduction

Venous thromboembolism (VTE) is one of the leading cardiovascular diagnoses worldwide [1]. Approximately, 50% of these events are associated with current or recent hospitalization [1]. Data from the national hospital discharge survey indicate that obese patients are at more than twice the risk of developing a VTE event during hospitalization compared to non-obese patients [2]. This is of growing concern given the prevalence of obesity in the United States. According to the Centers for Disease Control and Prevention, as of the year 2020, nearly 32% of the population had a body mass index (BMI) ≥ 30 kg/m^2^ [3].

To reduce the risk of VTE, prophylactic enoxaparin is routinely utilized among hospitalized patients. Standard prophylactic enoxaparin dosing consists of a fixed dose regimen of 40 mg administered subcutaneously once daily. This is contrary to therapeutic enoxaparin, which utilizes weight-based dosing, often administered as a twice daily (BID) injection. Standard prophylactic enoxaparin regimens may be inadequate for morbidly obese patients secondary to alterations in drug clearance and the volume of distribution (Vd) [4]. Enoxaparin’s Vd is nearly equivalent to plasma volume. As body weight increases, there is a non-linear increase in plasma volume. Additionally, alterations in enoxaparin clearance occur due to increased kidney mass and perfusion. These alterations have been noted to result in a non-linear increase in drug clearance [4]. Given these alterations, standard enoxaparin dosing may be inadequate for morbidly obese patients. To confirm the appropriate dosing of enoxaparin, anti-factor Xa (anti-Xa) testing has been utilized to monitor and adjust dosing in special populations, including obesity. However, despite its use, there currently is no consensus on anti-Xa treatment targets for the purpose VTE prophylaxis with enoxaparin.

The 2018 American Society of Hematology VTE prophylaxis guidelines identified enoxaparin dosing in obesity as a research priority [1]. Previous studies have assessed high-intensity fixed dosing and weight-based enoxaparin regimens compared to standard prophylactic dosing [5,6]. These studies have demonstrated improved attainment of target anti-Xa levels, as well as reductions in VTE events [5,6]. At the present time, there are limited data comparing high-intensity, fixed-dosing, and weight-based regimens to determine the optimal dosing strategy. Thus, the objective of this study was to assess anti-Xa target attainment between two high-intensity, fixed-dose enoxaparin regimens and a high-intensity, weight-based enoxaparin regimen.

## 2. Materials and Methods

This study was a retrospective chart review of morbidly obese patients receiving enoxaparin at one academic medical center between July 2018 and August 2021. The study protocol was approved by the Institutional Review Board at the University of Nebraska Medical Center. Patients were identified utilizing records of orders for enoxaparin and anti-Xa levels among patients with a BMI ≥ 40 kg/m^2^. Data were extracted from electronic medical records.

This study included adult (age ≥ 19 years) patients that received prophylactic enoxaparin at a dose of 40 mg BID, 60 mg BID, or 0.5 mg/kg BID and had a steady-state, anti-Xa peak level drawn during their admission. These dosing regimens were selected based on institutional practice. At the time of the study, no local guidelines existed to guide dosing practices and this was left to the physician’s discretion. Steady-state, peak anti-Xa levels had to be appropriately drawn 4–6 h after the administration of a minimum of three enoxaparin doses. Anti-Xa levels were measured using Instrumentation Laboratory ACL TOP 500 CTS. Patients were excluded if they had renal dysfunction (CrCl < 30 mL/min), active malignancy, pregnancy, bleeding, or thrombosis present at admission, thrombocytopenia (platelet ≤ 50 × 10^3^), coagulopathy, or receipt of therapeutic anticoagulation prior to the initial anti-Xa level.

The primary outcome for this study was the rate of target anti-Xa attainment. The target anti-Xa level was defined as 0.2–0.5 IU/mL. This range was determined based on a literature review, as well as institutional practice [6,7,8]. Secondary outcomes included the rate of VTE, major bleeding, subtherapeutic levels, supratherapeutic anti-Xa levels, and need for dose adjustment. Major bleeding was defined as a hemoglobin drop ≥ 2 g/dL requiring the discontinuation of enoxaparin or receipt of ≥2 units of blood products. This definition is a modified version of the Internation Society of Thrombosis and Hemostasis definition of major bleeding in non-surgical patients [9].

Categorical variables are reported as counts and percentages. Continuous variables are reported as means with standard deviation (SD). Categorical demographics variables were analyzed utilizing using Pearson’s Chi-squared test, and continuous variables were compared using Student’s *t* test. An ANCOVA was used for the multiple group analysis of continuous variables when appropriate. Primary and secondary outcomes were assessed utilizing univariate and multivariate logistic regression (adjusted for confounders when appropriate). Multivariate logistic regression models were constructed utilizing a forward-stepwise parameter selection with a p-threshold set at 0.2 for model inclusion. Secondary outcomes were assessed utilizing logistic regression. Clinical covariates were retained in the model if the *p* value was <0.1 and retention improved the overall model fitness by Akaike information criterion [10]. All statistical analyses were performed using Intercooled Stata, version 14.2 (College Station, TX, USA: StataCorp LP.) and Prism GraphPad, version 9 (GraphPad Software, San Diego, CA, USA, www.graphpad.com).

## 3. Results

### 3.1. Cohort Characteristics

**Figure 1:**
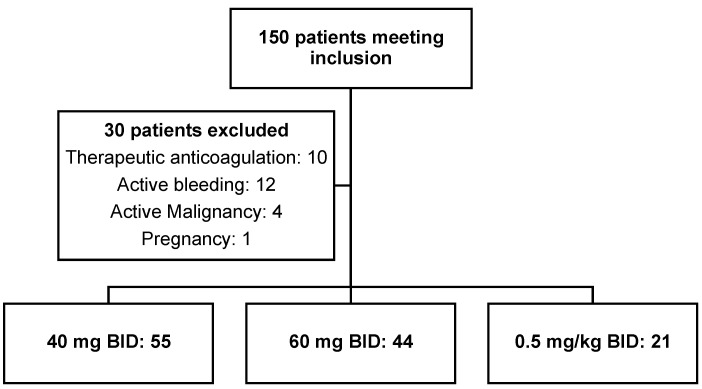
Patient Screening.

A total of 150 consecutive patients were screened for inclusion in this study, with 120 patients meeting inclusion criteria. Reasons for exclusion are included in Figure 1. In total, 55 patients received enoxaparin 40 mg BID, with 44 patients and 21 patients receiving 60 mg BID and 0.5 mg/kg BID, respectively. The mean (SD) patient weight was 166.7 (37.4) kg with a mean (SD) BMI of 56.8 (12.0) kg/m^2^. The mean (SD) admission weight was significantly lower among patients receiving 40 mg BID compared to 60 mg BID (153.7 (32.6) kg vs. 178.9 (34.3) kg, respectively, *p* = 0.0015). Patients in the 40 mg BID arm had a significantly lower mean (SD) BMI compared to both the 60 mg (51.5 (9.8) kg/m^2^ vs. 61.6 (10.7) kg/m^2^, respectively, *p* < 0.0001) and 0.5 mg/kg BID arms (51.5 (9.8) kg/m^2^ vs. 60.9 (14.5) kg/m^2^, respectively, *p* = 0.0032). The mean dose among patients receiving 0.5 mg/kg BID was 85 mg with a range of 50–150 mg. The full patient demographics can be found in Table 1. No missing data were present in this cohort.

### 3.2. Univariate Analysis: Anti-Xa Target Attainment

The results of a univariate analysis can be found in Table 2 (A,B). A significant difference in anti-Xa target attainment was found between 40 mg BID and 60 mg BID (29.1% vs. 54.5%, OR 2.9; *p* = 0.01). A significant difference was also found between patients receiving 40 mg BID and 0.5 mg/kg BID (29.1% vs. 90.5%, OR 23.2; *p* < 0.0001).

### 3.3. Multivariate Analysis: Anti-Xa Target Attainment

In the multivariate logistic regression model, the following clinical covariates were included: age, weight, baseline hemoglobin, acute infection, and heart failure. The results of the multivariate logistic regression were consistent with those of the univariate analysis. When adjusting for these confounders, anti-Xa target attainment was 4.1 times more likely among patients receiving 60 mg BID and 40.6 times more likely among patients receiving 0.5 mg/kg BID compared to 40 mg BID. The complete results of the multivariate analysis are presented in Table 3.

### 3.4. Secondary Outcomes

VTE events were rare in this cohort. A single pulmonary embolism was identified in a patient receiving 60 mg BID, which occurred in the setting of a therapeutic anti-Xa level. Major bleeding occurred in six patients in this cohort. There was no significant difference in the incidence of major bleeding events between dosing regimens (*p* = 0.24). Five of these bleeding events were related to recent surgery or other invasive procedures. One GI bleed occurred in a patient receiving 60 mg BID with a supratherapeutic anti-Xa level (anti-Xa: 0.51). Of the remaining bleeding events, two occurred in the setting of a therapeutic anti-Xa level and three occurred in patients with subtherapeutic anti-Xa levels.

The majority of non-therapeutic anti-Xa levels were the result of subtherapeutic levels. The incidence of subtherapeutic anti-Xa levels was significantly increased among those receiving 40 mg BID compared to 60 mg BID (69.1% vs. 40.9%, OR 0.31, *p* = 0.006) and 0.5 mg/kg BID (69.1% vs. 4.8%, OR 0.02, *p* < 0.005). Supratherapeutic anti-Xa levels were rare and there was no significant difference between dosing regimens (*p* = 0.68). Dose adjustments were more common among patients receiving 40 mg BID compared to 60 mg BID (69.1% vs. 36.4%, OR 0.26, *p* = 0.001) and 0.5 mg/kg BID (69.1% vs. 14.3%, OR 0.07, *p* < 0.005). Additional secondary outcomes can be found in Table 4.

## 4. Discussion

The current evidence supports high-intensity dosing of enoxaparin for VTE prevention in morbidly obese patients. However, evidence to inform optimal enoxaparin dosing is limited. Our study demonstrated a significant improvement in anti-Xa target attainment with 60 mg BID and 0.5 mg/kg BID compared to 40 mg BID. Weight-based dosing outperformed fixed dosing regimens in this study. There was no evidence of increased major bleeding or supratherapeutic anti-Xa levels with these high-intensity regimens. This study demonstrates that a high-intensity, weight-based enoxaparin regimen may be preferrable to high-intensity, fixed dosing regimens to optimize anti-Xa target attainment among morbidly obese patients. Additional studies are necessary to assess the impact that dosing has on clinical outcomes, such as VTE and major bleeding.

The findings of this study are consistent with the results of multiple previous studies [5,6,11]. A study by Frederiksen et al. demonstrated the presence of a strong negative correlation (R^2^ = 0.63) between anti-Xa activity and body weight among patients receiving a single dose of enoxaparin 40 mg [11]. Freeman et al. demonstrated the significance of this negative correlation in practice through a study assessing anti-Xa target attainment (0.2–0.5 IU/mL) in patients with a BMI ≥ 40 kg/m^2^ [6]. In their study, 82% of patients receiving enoxaparin 40 mg daily had a subtherapeutic anti-Xa level. These investigators assessed two additional dosing regimens, 0.4 mg/kg daily and 0.5 mg/kg daily. Compared to 40 mg daily, both 0.4 mg/kg daily (18% vs. 64%, *p* < 0.001) and 0.5 mg/kg daily (18% vs. 87%, *p* < 0.001) were associated with significant improvements in target anti-Xa levels [6]. While these improvements in anti-Xa target attainment are promising, they act as a surrogate marker for clinical outcomes. 

Wang et al. conducted a study assessing high-dose (enoxaparin 40 mg twice daily or heparin 7500 units three times daily) VTE prophylaxis versus standard-dose (enoxaparin 40 mg once daily or heparin 5000 units three times daily) VTE prophylaxis to identify the impact of dosing on clinical outcomes [5]. They assessed the rate of VTE and bleeding events among 9241 patients with a weight ≥ 100 kg, 43% of whom had a BMI ≥ 40 kg/m^2^. High-dose VTE prophylaxis was associated with a reduction in VTE events compared to standard dose prophylaxis (0.77% vs. 1.48%, *p* = 0.05) without an increase in major bleeding (7.18% vs. 8.44%, *p* = 0.15) among patients with a BMI ≥ 40 kg/m^2^ [5]. Additional studies are necessary to confirm the impact of high-dose VTE prophylaxis strategies on clinical outcomes and to validate an anti-Xa target to guide prophylactic enoxaparin dosing for obese patients.

This study’s limitations include the fact that it is a retrospective analysis subject to biases and risks of misclassification. The study site did not provide any enoxaparin dosing guidance for morbidly obese patients at the time of this study. This introduces the opportunity for treatment bias due to variable utilization of these dosing strategies between patient populations. This study was underpowered to detect a difference in clinical outcomes and thus utilized anti-Xa levels, which is a surrogate marker for clinically relevant outcomes. A validated target for anti-Xa levels for VTE prophylaxis has not been established, requiring a target range to be defined based on a literature review and institutional practice. Finally, we only assessed initial anti-Xa levels and did not evaluate subsequent levels to evaluate for evidence of drug accumulation.

Based on the results of this study, the investigators suggest the use of high-dose VTE prophylaxis strategies (i.e., 60 mg BID, 0.5 mg/kg BID) in clinical practice for morbidly obese patients with normal renal function (CrCl ≥ 30 mL/min). Peak anti-Xa levels (4 h post-dose) should be monitored after the receipt of three doses of enoxaparin to ensure attainment of target anti-Xa levels.

## 5. Conclusions

In this study, enoxaparin 60 mg BID and 0.5 mg/kg BID improved anti-Xa target attainment compared to 40 mg BID. These dosing strategies also significantly reduced the incidence of subtherapeutic levels without increasing supratherapeutic levels or major bleeding events. Further, in this study, weight-based dosing outperformed fixed dosing strategies. Additional studies are necessary to determine the impact of high-intensity dosing regimens on clinical outcomes.

## Figures and Tables

**Table 1:** Patient Characteristics.

	Overall (N = 120)	40 mg BID (*n* = 55)	60 mg BID (*n* = 44)	0.5 mg/kg BID (*n* = 21)	*p*-Value
Age (years), mean (SD)	50.8 ± 13.1	50.8 ± 13.8	50.5 ± 13.1	51.5 ± 11.6	0.96
Male, *n* (%)	63 (52.5%)	31 (56.4)	18 (40.9)	8 (38.1)	0.2
Race/Ethnicity, *n* (%)					
Caucasian	88 (73)	39 (71)	36 (82)	13 (62)	0.2
African American	18 (15)	8 (15)	6 (14)	4 (19)	0.84
American Indian	18 (15)	8 (15)	6 (14)	4 (19)	0.84
Other	9 (8)	7(13)	1 (2)	1 (5)	0.12
Unknown	2 (2)	-	-	2 (10)	-
Weight (kg), mean (SD)	166.7 ± 37.4	153.7 ± 32.6	178.9 ± 34.3	175.4 ± 45.4	0.0015 *
BMI (kg/m^2^), mean (SD)	56.8 ± 12.0	51.5 ± 9.8	61.6 ± 10.7	60.9 ± 14.5	<0.0001 * 0.0032 **
Level of Care, *n* (%)					
Progressive Care	15 (13)	7 (13)	6 (14)	2 (10)	0.89
Critical Care	43 (36)	21 (38)	16 (36)	6 (29)	0.73
Serum Creatinine, mean (SD)	0.90 ± 0.37	0.90 ± 0.32	0.96 ± 0.46	0.77 ± 0.21	0.14
Creatinine Clearance, mean (SD)	150.5 ± 62.8	142.9 ± 52.9	149.5 ± 60.2	172.4 ± 86.4	0.19
Platelets, mean (SD)	237 ± 85	243 ± 96	232 ± 83	233 ± 51	0.77
Hemoglobin, mean (SD)	12.4 ± 2.2	13.0 ± 2.3 *	11.9 ± 1.8 *	11.9 ± 2.5	0.02 *
Heart Failure, *n* (%)	30 (25)	8 (15)	16 (36)	6 (29)	0.04
Respiratory Comorbidity, *n* (%)	68 (57)	26 (47)	27 (61)	15 (71)	0.12
Surgery, *n* (%)	51 (43)	26 (47)	17 (39)	8 (38)	0.62
History of VTE, *n* (%)	3 (3)	0 (0)	3 (7)	0 (0)	0.07

* = comparator groups (40 mg vs 60 mg). ** = comparator groups (40 mg vs 0.5 mg/kg). BID, twice daily; BMI, body mass index; IQR, interquartile range; kg, kilogram; kg/m^2^, kilogram per square meter; SD, standard deviation; and VTE, venous thromboembolism. Note: one-way ANOVA was used for between group comparison (40 mg vs. 60 mg vs. 0.5 mg/kg). Categorical comparisons were made via Chi-squared test.

**Table 2:** **A.** Anti-Xa target attainment (40 mg vs. 60 mg; univariate analysis). **B.** Anti-Xa target attainment (40 mg vs. 0.5 mg/kg; univariate analysis).

**(A)**				
	40 mg BID *n* = 55	60 mg BID *n* = 44	OR	*p*-value
Target Anti-Xa Attainment, *n* (%)	16 (29.1)	24 (54.5)	2.9	0.01
**(B)**				
	40 mg BID *n* = 55	0.5 mg/kg BID *n* = 21	OR	*p*-value
Target Anti-Xa Attainment, *n* (%)	16 (29.1)	19 (90.5)	23.16	<0.0001

BID, twice daily; OR, odds ratio.

**Table 3:** Anti-Xa target attainment (multivariate analysis).

Variable *	aOR		95% CI		SE		*p*-Value	
Enoxaparin Dose (Control: 40 mg BID)	60 mg BID	0.5 mg/kg BID	60 mg BID	0.5 mg/kg BID	60 mg BID	0.5 mg/kg BID	60 mg BID	0.5 mg/kg BID
	4.05	40.55	1.6–10.3	6.96–236.16	1.92	36.46	0.003	<0.005
Weight (kg)	0.98		0.97–0.997		0.006		0.078	
Constant	3.78		0.56–25.7		3.7		0.17	

* Variables evaluated in the model included dose of enoxaparin, age, weight, baseline hemoglobin, presence of acute infection, and presence of heart failure. A *p*-value of 0.2 was used for inclusion and further evaluation. Abbreviations: aOR, adjusted odds ratio; BID, twice daily; CI, confidence interval; and SE, standard error.

**Table 4:** Secondary outcomes (univariate analysis).

	40 mg BID *n* = 55	60 mg BID *n* = 44	0.5 mg/kg BID *n* = 21	*p*-Value
DVT, *n* (%)	0 (0)	0 (0)	0 (0)	-
PE, *n* (%)	0 (0)	1 (2.3)	0 (0)	-
Major Bleeding, *n* (%)	1 (1.8)	4 (9.1)	1 (4.8)	0.24
Control: 40 mg BID	-	OR 5.4 *	OR 2.7 *	
	*p*-Value	0.14 *	0.49 *	
Need for dose adjustment, *n* (%)	38 (69.1)	16 (36.4)	3 (14.3)	<0.005
Control: 40 mg BID	-	OR 0.26 *	OR 0.07 *	
	*p*-Value	0.001 *	<0.005 *	
Subtherapeutic anti-Xa, *n* (%)	38 (69.1)	18 (40.9)	1 (4.8)	<0.005
Control: 40 mg BID	-	OR 0.31 *	OR 0.02 *	
	*p*-Value	0.006 *	<0.005 *	
Supratherapeutic anti-Xa, *n* (%)	1 (1.8)	2 (4.5)	1 (4.8)	0.68
Control: 40 mg BID	-	OR 2.57 *	OR 2.7 *	
	*p*-Value	0.45 *	0.49 *	
Mortality, *n* (%)	5 (9.1)	2 (4.5)	0 (0)	-

* Between group comparison OR and *p*-values refer to comparisons to control (40 mg BID). BID, twice daily; DVT, deep vein thrombosis; OR, Odds Ratio; PE, pulmonary embolism; and VTE, venous thromboembolism.

## Data Availability

Data are contained within the article.
